# Primary Cardiac Diffuse Large B-Cell Lymphoma: Diagnostic Journey of Undifferentiated Right Atrial Mass

**DOI:** 10.1016/j.jaccas.2024.102924

**Published:** 2024-12-18

**Authors:** Nitish K. Dhingra, Dambuza Nyamande, Abdulaziz M. Alhothali, Abdullah H. Ghunaim, Robert J. Cusimano

**Affiliations:** Division of Cardiac Surgery, Toronto General Hospital, University of Toronto, Toronto, Ontario, Canada

**Keywords:** cardiac tumor, lymphoma, percutaneous biopsy, transvenous biopsy

## Abstract

Undifferentiated cardiac tumors represent a diagnostic dilemma that requires meticulous workup. We present the case of a 67-year-old woman with a right atrial mass that was investigated with multiple modalities before being diagnosed as a cardiac lymphoma. The role of transvenous and/or percutaneous biopsy for guiding diagnosis and management is emphasized.

## Graphical Abstract

**Figure:**
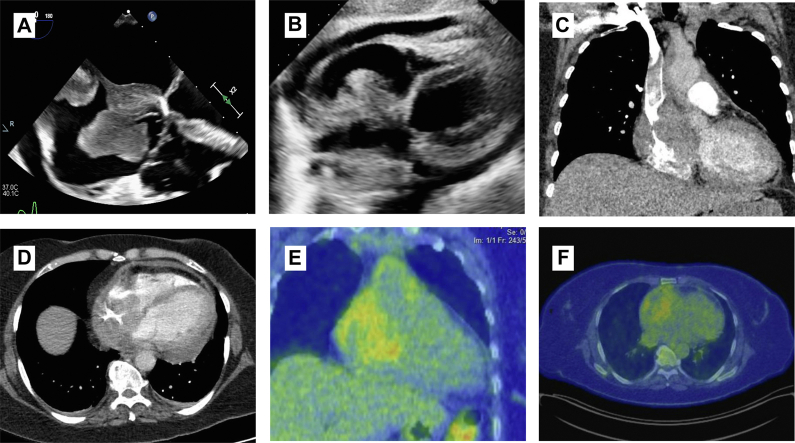

A 67-year-old woman presented with a subacute history of dyspnea, pleuritic chest pain, and fever of approximately 38 °C. There was no orthopnea, paroxysmal nocturnal dyspnea, weight loss, or palpitations. The patient was euvolemic on examination and did not have any evidence of clinical lymphadenopathy. The patient’s past medical history was significant for hypertension, type 2 diabetes, dyslipidemia, and gastroesophageal reflux.

An initial chest x-ray showed a mildly enlarged cardiac silhouette, and initial blood workup showed a mildly elevated initial lactate dehydrogenase at 238 U/L (100-220 U/L). Blood cultures were negative. Electrocardiogram demonstrated mild anterolateral ST-segment depressions in leads V_4_ to V_6_. Given these findings, an echocardiogram was performed and demonstrated depressed left ventricular systolic function (left ventricular ejection fraction of 35%-45%) and a large right atrial tumor (Figures 1A and 1B, Video 1). A chest computed tomography (CT) confirmed the presence of the right atrial mass extending into the superior vena cava with associated superior vena cava narrowing and involvement of the inferior vena cava, aortic root, left atrium, and pericardium (Figures 1C and 1D). A cardiac magnetic resonance imaging added no new information. A positron emission tomography demonstrated mild avidity of the right atrial tumor (maximum standardized uptake value of 4.0), along with mildly active supraclavicular lymph nodes (Figures 1E and 1F). Coronary angiography was normal.

**Figure 1:**
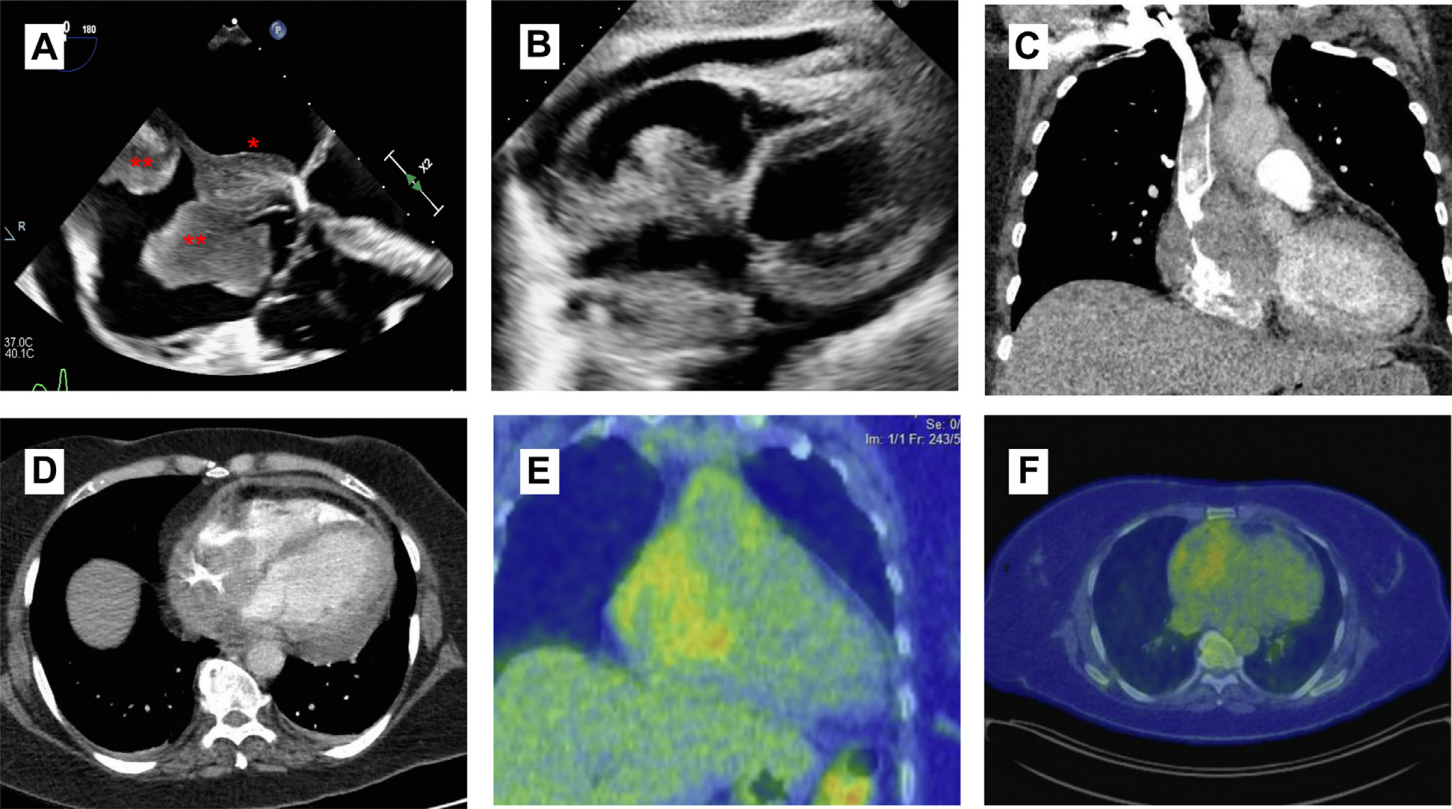
Imaging Results Right atrial mass demonstrated on multiple imaging modalities including echocardiography (A and B), computed tomography (CT) (C and D), and positron emission tomography (E and F). On transesophageal echocardiography (B), the multifocal mass is demonstrated (∗∗) along with a component crossing the interatrial septum (∗). The patient was ultimately diagnosed with a diffuse large B-cell lymphoma after a CT-guided biopsy of the tumor and appropriately initiated on chemotherapy with R-CHOP (rituximab, cyclophosphamide, doxorubicin, vincristine, and prednisone).

Cardiac surgery was consulted for an operation. After discussion, a multidisciplinary decision was made to proceed with transvenous right atrial tumor biopsy and ultrasound-guided core-needle biopsy of the scalene lymph nodes, which both failed to yield diagnostic tissue. Therefore, a CT-guided percutaneous biopsy of the right atrial mass was performed, which yielded a tissue diagnosis of diffuse large B-cell lymphoma. The patient was treated with R-CHOP (rituximab, cyclophosphamide, doxorubicin, vincristine, and prednisone) with a good response.

Tissue diagnosis of any tumor is mandatory for prognosis and treatment. This is especially true in cardiac tumors. Indeed, the differential for an undifferentiated right atrial mass is broad and includes thrombus, myxoma, sarcoma, secondary metastatic disease, and rarely lymphoma, among other potential diagnoses.^1^ Given the difficulty in obtaining tissue and the gravity of a malignant tumor of the heart,^2^ especially when locally advanced such as in our patient, a plan for biopsy is imperative. Right-sided lesions readily lend themselves to transvenous biopsy, but this modality does not always yield diagnostic tissue. A total body survey looking for other more easily obtainable tissue should always be sought. When these fail, we have been able to perform CT-guided biopsy^3^ with good results. If this was nondiagnostic, an excisional biopsy (in this case impossible) or open biopsy through a small incision performed off-pump would be the last step. Using the stepwise strategy, the diagnosis of primary cardiac lymphoma was made and led to chemotherapy with a hope for nonsurgical cure.

Primary cardiac lymphomas represent <2% of all cardiac tumors, most commonly in the right heart. Almost half of patients present with congestive heart failure, and it is unclear if that is related to obstruction or contractility changes in patients with primary cardiac lymphoma.^4^ The reason for diminished ventricular function in our patient with normal coronaries and isolated atrial involvement is unclear. Outcomes have improved with 5-year survival going from 0% to over 80% with therapy.^5^

## Funding Support and Author Disclosures

The authors have reported that they have no relationships relevant to the contents of this paper to disclose.

## Appendix

Video 1 Transesophageal echocardiography demonstrating a right atrial mass.
